# Supplementary material for: Antimicrobial properties of a multi-component alloy
Source: Sci Rep. 2022 Dec 11;12:21427. doi: 10.1038/s41598-022-25122-4 (PMC9741758; doi:10.1038/s41598-022-25122-4)
Supplement: Supplementary file 1 — Supplementary Information. [file 41598_2022_25122_MOESM1_ESM.docx]

# Supplemental Material for:

**Anti-microbial properties of a multi-component alloy**

Anne F. Murray^1,2^, Daniel Bryan^1^, David A. Garfinkel^3^, Cameron S. Jorgensen^3^, Nan Tang^3^, WLNC Liyanage^3^, Eric A. Lass^3^, Ying Yang^4^, Philip D. Rack^3^, Thomas G. Denes^1^, and Dustin A. Gilbert^3,5*^

^1^Department of Food Science, University of Tennessee, Knoxville, Tennessee 37996

^2^Department of Ecology and Evolutionary Biology, University of Tennessee, Knoxville, Tennessee 37996

^3^Department of Material Science, University of Tennessee, Knoxville, Tennessee 37996

^4^ Oakridge National Laboratory, Material Science and Technology Division, Oakridge, Tennessee, 37831

^5^ Department of Physics and Astronomy, University of Tennessee, Knoxville, Tennessee 37996

* Corresponding author (D.A.G.): E-mail: dagilbert@utlk.edu

**
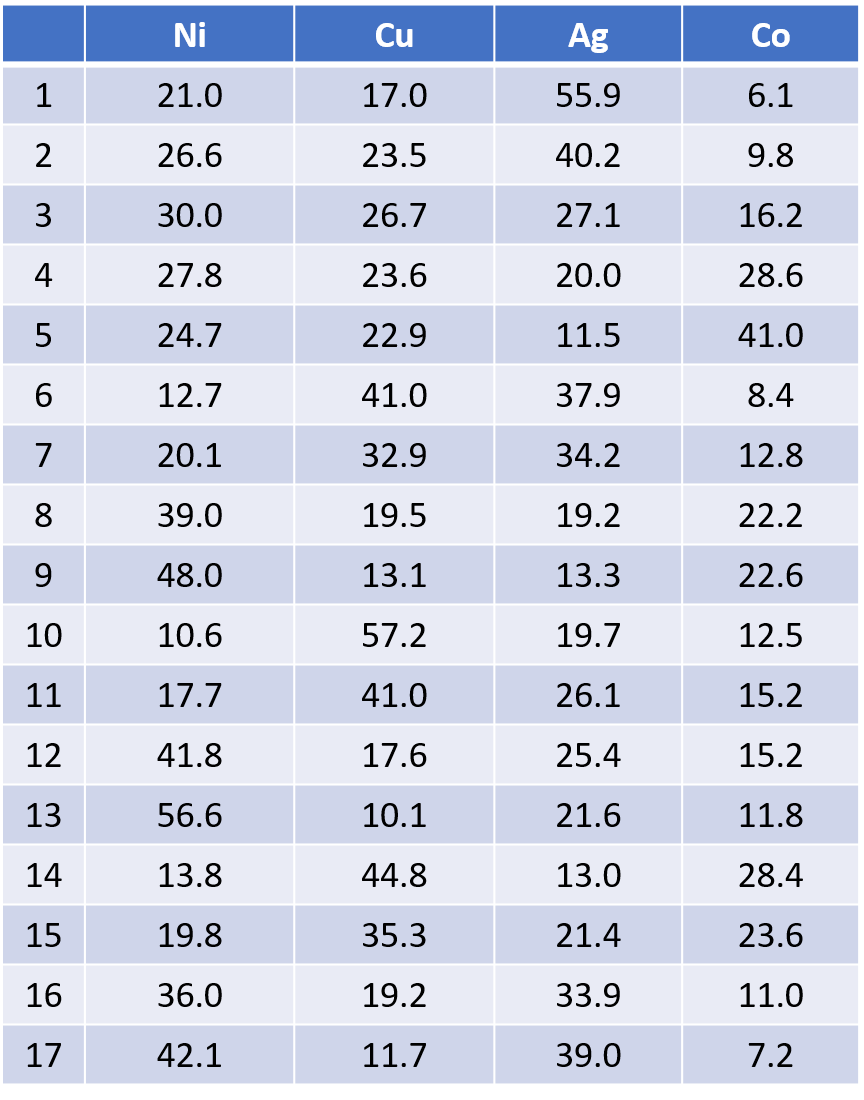
**
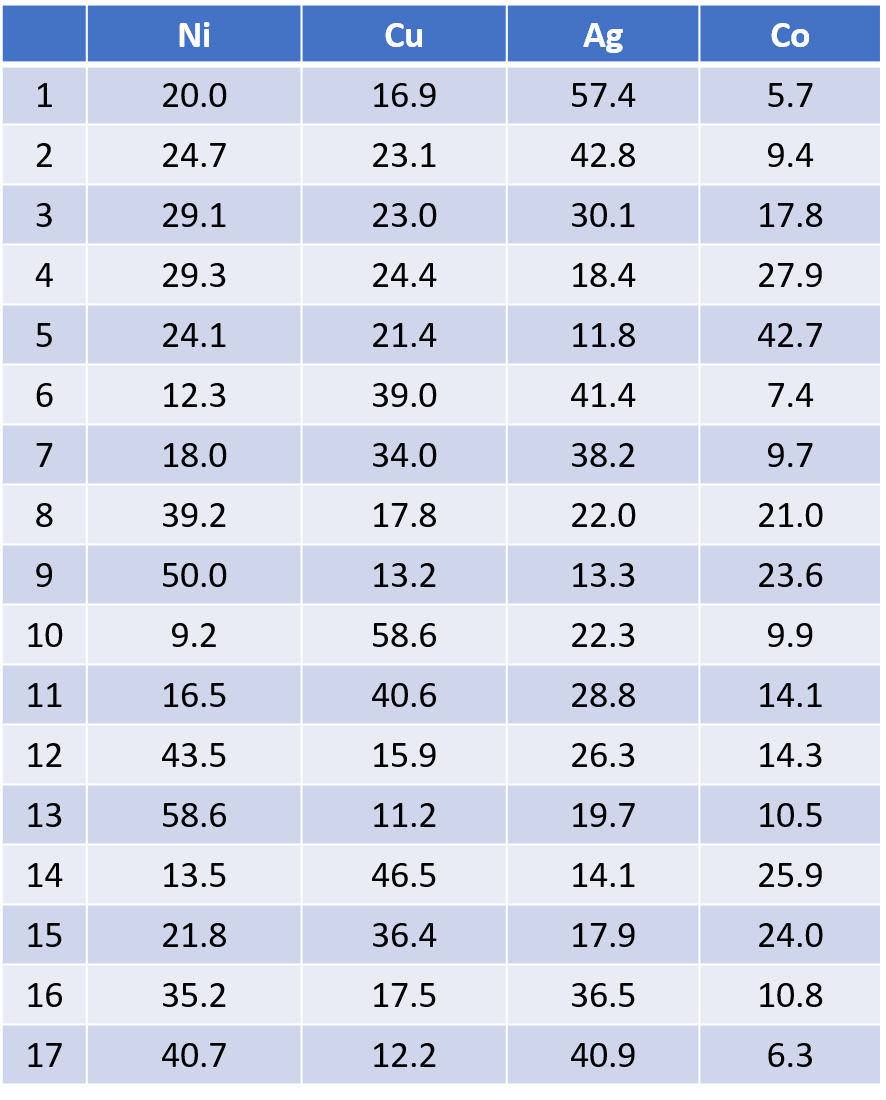


a

b

**Table S1**. Metal composition of each chip as percentage of the wafer surface area determined by X-ray diffraction of (a) as-grown and (b) annealed.

**
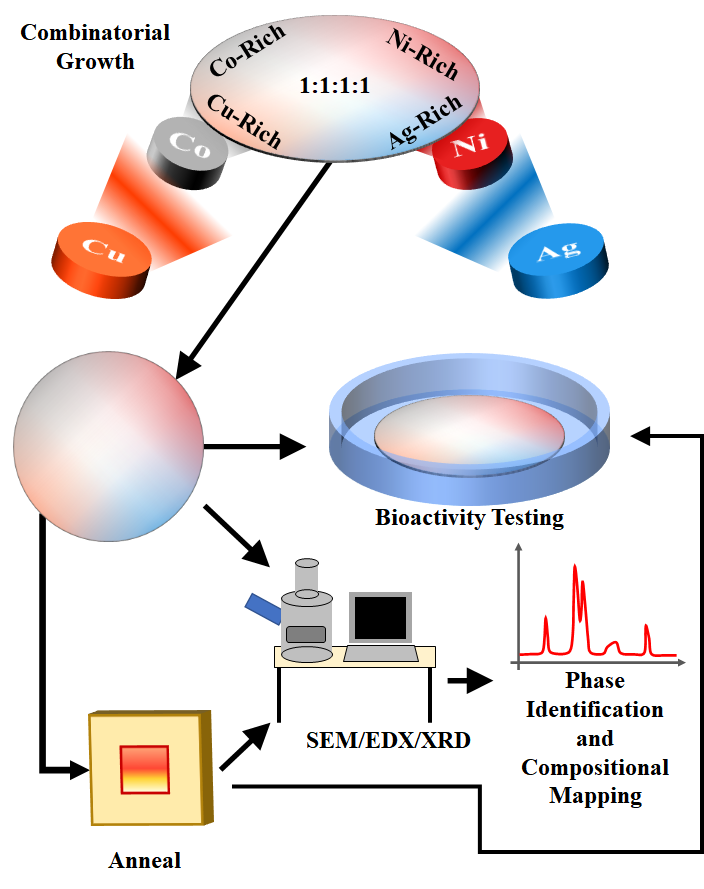
**

**Figure S1** Illustrative diagram of the film synthesis and testing procedure

**
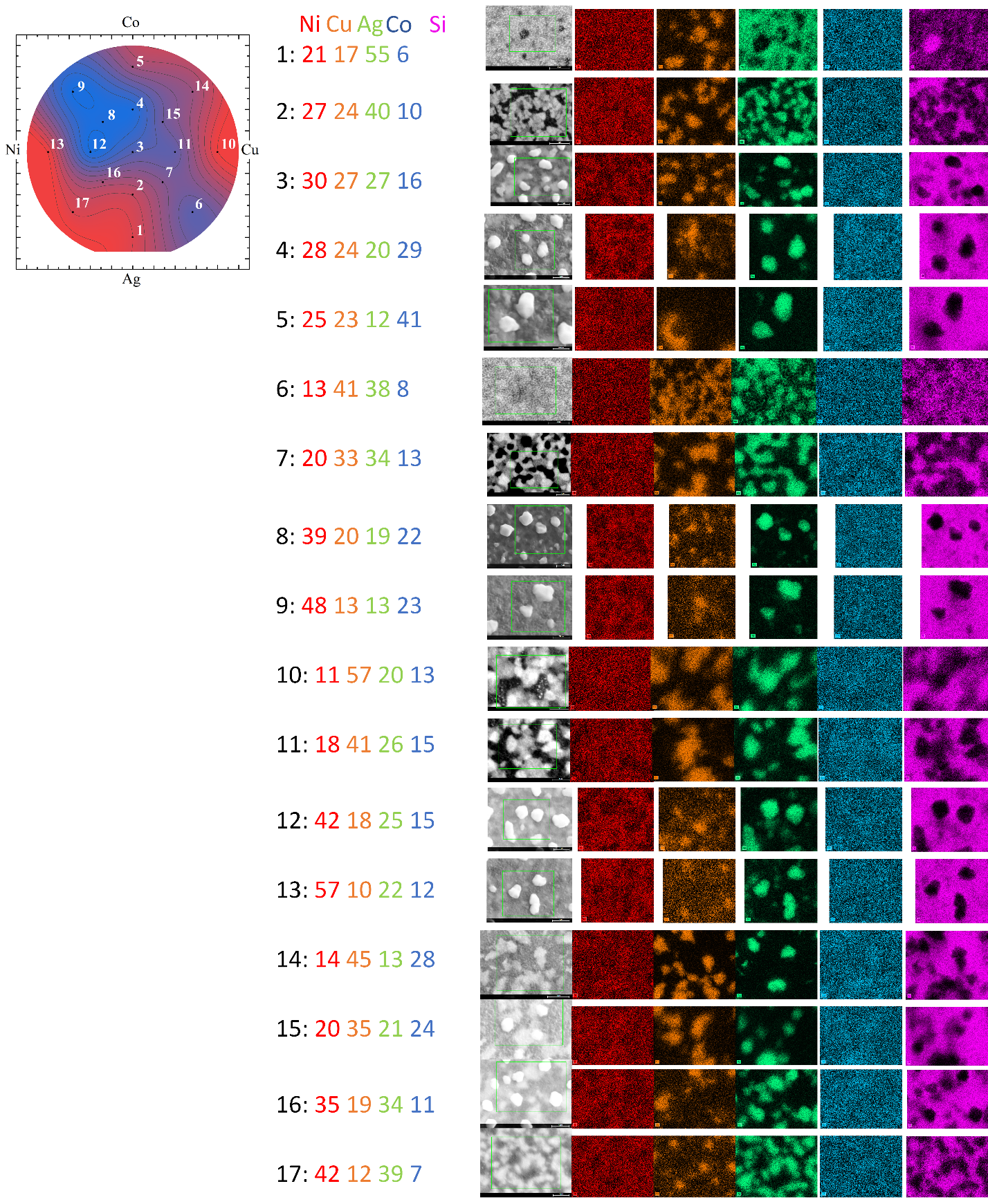
**

**Figure S2** Complete collection of EDX images, with position on the wafer and composition indicated in the left column.

**
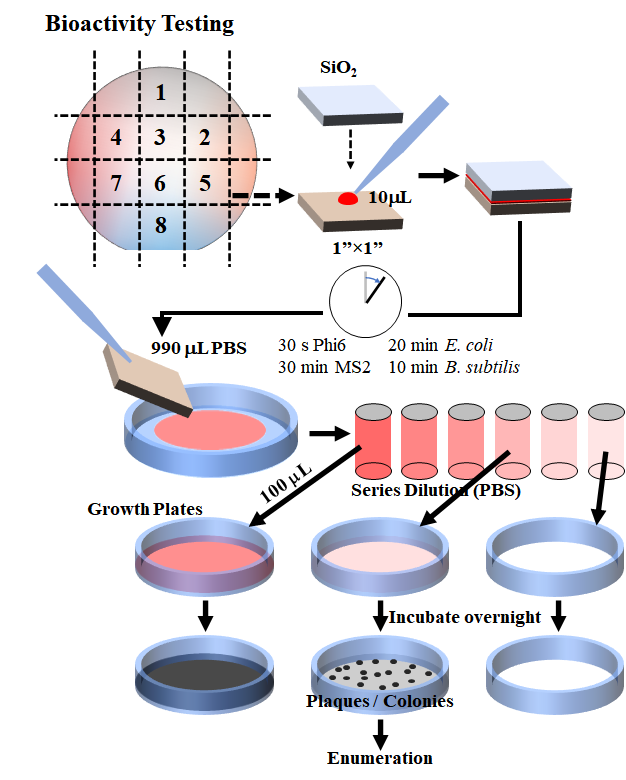
**

**Figure S3** Illustrative diagram of the biological testing procedure.

| Organism | Co | Ni | Ag | Cu |  |
| --- | --- | --- | --- | --- | --- |
| Phi6 | 1.2 | 1.2 | 1.4 | 6.9 |  |
| MS2 | 0.8 | 0.5 | 0.2 | 7.1 |  |
| *E. coli* | 0.3 | 0.2 | 0.7 | 5.9 |  |
| *B. subtilis* | 0.7 | 1.2 | 1.0 | na |  |
|  |  |  |  |  |  |

**Figure S4.** Log reduction of a) Phi6, b) MS2, c) *E. coli*, and d) *B. subtilis* on individual bioactive metals. An asterisk (*) indicates a significant difference (*p*<0.05) from the negative control. Table indicates log-reductions values. na indicates no organisms were recovered.

| Organism | Pd | Ti | Zn | Cu (+) |  |
| --- | --- | --- | --- | --- | --- |
| Phi6 | 0.2 | 1.2 | 0.5 | na |  |
| MS2 | 3.3 | 0.9 | 2.1 | na |  |
| *E. coli* | 0.2 | 0.4 | 0.2 | na |  |
| *B. subtilis* | - | - | 1.0 | 5.6 |  |
|  |  |  |  |  |  |
|  |  |  |  |  |  |

**Figure S5.** Log reduction of a) Phi6, b) MS2, c) *E. coli*, and d) *B. subtilis* on individual metals. Table indicates log-reductions values. No Phi6, MS2 or *E. coli* were detected on the Cu control but *B. subutilis* experienced a 5.6 log reduction. na indicates no organisms were recovered.

| Organism | Br  low | Br high | CuO | Cu_2_O |  |  |
| --- | --- | --- | --- | --- | --- | --- |
| Phi6 | na | na | 6.5 | 2.8 |  |  |
| MS2 | 4.0 | 5.3 | 4.8 | 4.9 |  |  |
| *E. coli* | na | 5.6 | na | 5.8 |  |  |
| *B. subtilis* | 4.5 | 5.2 | na | 4.5 |  |  |
|  |  |  |  |  |  |  |
|  |  |  |  |  |  |  |

**Figure S6.** Log reduction of a) Phi6, b) MS2, c) *E. coli*, and d) *B. subtilis* on brass and copper oxides. Table indicates log-reductions values. na indicates no colonies or plaques were recovered on the treatment. No Phi6, *E. coli* or *B. subtilis* was detected on the Cu control except for MS2 which showed a log reduction of 6.1 and 5.8 respective assays.

| Organism | Currency | Cu (+) |
| --- | --- | --- |
| Phi6 | 3.2 | na |
| MS2 | 2.0 | 5.0 |
| *E. coli* | 4.5 | na |
| *B. subtilis* | 0.5 | na |

**Figure S7.** Log reduction of a) Phi6, b) MS2, c) *E. coli*, and d) *B. subtilis* on currency prototype. An asterisk (*) indicates a significant difference (*p*<0.05) from the negative control. Table indicates log-reductions values. No Phi6, *E. coli*, *B. subtilis* were detected on the Cu control but MS2, experienced a 5.0 log reduction.

| Chip # | Phi6 | MS2 | *E. coli* | *B. subtilis* |
| --- | --- | --- | --- | --- |
| 1 | 1.8 | 1.9 | 2.1 | 2.7 |
| 2 | 6.0 | 4.9 | 6.4 | 5.7 |
| 3 | 3.1 | 4.0 | 5.1 | 5.3 |
| 4 | 2.2 | 2.5 | 2.8 | 4.3 |
| 5 | 6.9 | 5.0 | 5.3 | 5.2 |
| 6 | 3.4 | 2.2 | 4.8 | 5.5 |
| 7 | 1.9 | 1.3 | 3.2 | 2.7 |
| 8 | 2.7 | 1.7 | 2.4 | 2.9 |

**Figure S8.** Means comparison of log-reduction of a) Phi6, b) MS2, c) *E. coli*, and d) *B. subtilis* on the as deposited chips. Different letters indicate significant differences (*p*<0.05). Table indicates log-reductions values. No Phi6 or *B. subtilis* was detected on the Cu control but MS2 and *E. coli*, experienced a 7.2 and 5.9 log reduction, respectively.

| Chip # | Phi6 | MS2 | *E. coli* | *B. subtilis* |
| --- | --- | --- | --- | --- |
| 1 | 2.1 | 2.0 | 1.5 | 1.8 |
| 2 | 6.2 | 6.8 | 6.1 | 5.8 |
| 3 | 2.3 | 3.6 | 5.5 | 1.3 |
| 4 | 3.2 | 2.4 | 2.1 | 0.2 |
| 5 | 3.9 | 6.4 | 6.0 | 4.8 |
| 6 | 4.0 | 5.3 | 6.1 | 0.4 |
| 7 | 4.0 | 1.9 | 2.1 | 0.3 |
| 8 | 3.3 | 2.9 | 2.2 | 0.7 |

**Figure S9.** Means comparison of log-reduction of a) Phi6, b) MS2, c) *E. coli*, and d) *B. subtilis* on the thin film annealed chips. Different letters indicate significant differences (*p*<0.05). Table indicates log-reductions values. No organisms were detected on the Cu control.
